# Biodistribution and radiation dosimetry of [^64^Cu]copper dichloride: first-in-human study in healthy volunteers

**DOI:** 10.1186/s13550-017-0346-4

**Published:** 2017-12-12

**Authors:** M.A. Avila-Rodriguez, C. Rios, J. Carrasco-Hernandez, J. C. Manrique-Arias, R. Martinez-Hernandez, F. O. García-Pérez, A. R. Jalilian, E. Martinez-Rodriguez, M. E. Romero-Piña, A. Diaz-Ruiz

**Affiliations:** 1 Unidad Radiofarmacia-Ciclotrón, División de Investigación, Facultad de Medicina, Universidad Nacional Autónoma de México, 04510 Cd.Mx, Mexico; 2 Departamento de Neuroquímica, Instituto Nacional de Neurología y Neurocirugía Manuel Velasco Suárez S.S.A, Ave. Insurgentes Sur No. 3877, 14269 Ciudad de México, Mexico; 3 Departamento de Medicina Nuclear, Instituto Nacional de Cancerología, 14080 Cd.Mx, Mexico; 4 Department of Nuclear Sciences and Applications, International Atomic Energy Agency (IAEA), Vienna, Austria

**Keywords:** Copper-64, [^64^Cu]CuCl_2_, Copper biodistribution, Radiation dosimetry, Theranostics

## Abstract

**Background:**

In recent years, Copper-64 (T_1/2_ = 12.7 h) in the chemical form of copper dichloride ([^64^Cu]CuCl_2_) has been identified as a potential agent for PET imaging and radionuclide therapy targeting the human copper transporter 1, which is overexpressed in a variety of cancer cells. Limited human biodistribution and radiation dosimetry data is available for this tracer. The aim of this research was to determine the biodistribution and estimate the radiation dosimetry of [^64^Cu]CuCl_2_, using whole-body (WB) PET scans in healthy volunteers. Six healthy volunteers were included in this study (3 women and 3 men, mean age ± SD, 54.3 ± 8.6 years; mean weight ± SD, 77.2 ± 12.4 kg). After intravenous injection of the tracer (4.0 MBq/kg), three consecutive WB emission scans were acquired at 5, 30, and 60 min after injection. Additional scans were acquired at 5, 9, and 24 h post-injection. Low-dose CT scan without contrast was used for anatomic localization and attenuation correction. OLINDA/EXM software was used to calculate human radiation doses using the reference adult model.

**Results:**

The highest uptake was in the liver, followed by lower and upper large intestine walls, and pancreas, in descending order. Urinary excretion was negligible. The critical organ was liver with a mean absorbed dose of 310 ± 67 μGy/MBq for men and 421 ± 56 μGy/MBq for women, while the mean WB effective doses were 51.2 ± 3.0 and 61.8 ± 5.2 μSv/MBq for men and women, respectively.

**Conclusions:**

To the best of our knowledge, this is the first report on biodistribution and radiation dosimetry of [^64^Cu]CuCl_2_ in healthy volunteers. Measured absorbed doses and effective doses are higher than previously reported doses estimated with biodistribution data from patients with prostate cancer, a difference that could be explained not just due to altered biodistribution in cancer patients compared to healthy volunteers but most likely due to the differences in the analysis technique and assumptions in the dose calculation.

## Background

Given its half-life and decay scheme, ^64^Cu (T_1/2_ = 12.7 h, 17.4% β^+^, 39% β^−^, 43.6% EC) has the potential to serve a dual role in the development of both molecular agents for PET imaging and radioimmunotherapy drugs in oncology [1]. In addition to the therapeutic potential of its beta decay, the emission of Auger electrons, associated with the electron capture decay, can also considerably contribute to the therapeutic effectiveness of this radionuclide provided its internalization to the cell nucleus. Over the last several years, ^64^Cu has emerged as a promising radionuclide for imaging and important advances have been achieved in preclinical and clinical applications, using a broad variety of biomolecules, especially receptor-specific peptide conjugates and other biologically relevant small molecules, monoclonal antibodies, and nanoparticles linked to suitable chelators [2]. In recent years, ^64^Cu in the chemical form of copper chloride ([^64^Cu]CuCl_2_) has been identified as a potential theranostic agent for PET imaging and targeted radionuclide therapy (TRNT) using the human copper transporter 1 (hCTR1) as a molecular target [3, 4].

Preclinical studies using [^64^Cu]CuCl_2_ as a theranostic agent in selected human cancer xenograft models in mice, such as glioblastoma multiforme (U-87MG) and malignant melanoma (B16F10, A375M), demonstrated its potential as a therapeutic agent [5, 6]. A case report study of Valentini et al. evaluated the potential of [^64^Cu]CuCl_2_ as a therapeutic agent in two patients with metastatic prostate and uterine cancer, respectively [7]. They observed a significant reduction in volume of lesions and an improvement in overall condition following a single cycle treatment with 3700 MBq of [^64^Cu]CuCl_2_. Future clinical applications of [^64^Cu]CuCl_2_ will require accurate dosimetric data, especially for therapeutic procedures; however, only limited human biodistribution data and organ dosimetry are currently available for this radionuclide in the chemical form of copper chloride. Capasso et al. obtained human biodistribution data from patients with prostate cancer and estimated radiation doses, but the main purpose of Capasso’s research was not to perform the dosimetry but to investigate the role of [^64^Cu]CuCl_2_ PET/CT in staging of prostate cancer [8]. The dosimetric calculations were performed with limited data points, which limit the confidence in the data curve-fitting for determining the time-integrated activity coefficients in source organs. They included seven patients acquiring emission scans of abdomen and pelvis at 10 min for all patients, while whole-body (WB) PET/CT scans at 1, 3, and 24 h were acquired in only two patients. To the best of our knowledge, there are not any reports on radiation dosimetry estimates based on normal biodistribution data from healthy volunteers.

The aim of the current research is to estimate the human radiation dosimetry of [^64^Cu]CuCl_2_, using biodistribution data obtained from serial WB PET/CT scans in healthy volunteers.

## Methods

### Preparation of [^64^Cu]CuCl_2_

High specific activity ^64^Cu in the chemical form of copper dichloride was produced at the Unidad Radiofarmacia-Ciclotrón, Facultad de Medicina, UNAM, via the ^64^Ni(p,n)^64^Cu reaction with 11-MeV protons, by methods previously reported [9, 10]. After radiochemical purification, the copper fraction was evaporated to dryness and recovered with 5 ml of physiological saline solution and sterilized by passing it through a 0.22-μm syringe filter (Millex-GV). The pH of the reconstituted solution of [^64^Cu]CuCl_2_ in physiological saline was around 5.5, with a typical apparent molar activity in the range of 150–700 GBq/μmol as determined by titration of [^64^Cu]CuCl_2_ with the chelator 2-S-(4-Aminobenzyl)-1,4,7-triazacyclononane-1,4,7-triacetic-acid (p-NH2-Bn-NOTA). Radionuclidic purity at the time of injection was > 99% as determined by gamma spectroscopy in a HPGe detector.

### Human subjects

Six healthy volunteers were included (3 women and 3 men; mean age ± SD, 54.3 ± 8.6 years; age range, 47–70 years; mean weight ± SD, 77.2 ± 12.4 kg; weight range, 60–97 kg). Volunteers were recruited at the Instituto Nacional de Neurología y Neurocirugía with the approval of the Bioethical Committee. Written informed consent was obtained from each subject. Prescreening consisted of a detailed review of medical history and a physical examination. Subjects with evidence of clinical disease or a history of organ removal surgery were excluded.

### PET/CT imaging

Imaging was performed with a Biograph mCT 20 PET/CT scanner (Siemens Medical Solutions, USA) at the Instituto Nacional de Cancerología. Before administration of the tracer a low-dose CT scan without contrast was acquired for anatomical localization and attenuation correction. After intravenous injection of [^64^Cu]CuCl_2_ (4.0 MBq/kg), three consecutive WB emission scans were acquired at 5, 30, and 60 min post-injection (p.i.). In order to keep the X-ray dose as low as possible, subjects remained motionless on the bed of the scanner until the first series of emission scans was completed, so that the same CT scan could be used for the 3 emission scans. Additional PET/CT scans were acquired at 5, 9, and 24 h p.i. The WB PET scans were acquired from the vertex to mid thighs, with 2–3 min per bed position. PET images were reconstructed using a 2D ordered subset expectation maximization (OSEM2D) algorithm, and were corrected for scatter and random events, dead time, and decay. Resulting voxels were stored in units of Bq/cm^3^. The verification of the cross-calibration between the PET scanner and the dose calibrator was performed by a uniform phantom filled with a ^18^F solution.

### Imaging analysis

All PET/CT images were archived in Digital Imaging and Communications in Medicine (DICOM) format and were analyzed using OsiriX MD Imaging Software (Pixmeo SARL, Bernex, Switzerland). Only organs with a percentage uptake greater than their mass percentage of total body weight [(*organ weight*/*total body weight*)×*100*] were considered as source organs. Liver, pancreas, kidneys, and bowels met this criterion. The rest of the injected activity was assumed to be homogeneously distributed over the rest of the organs and tissues and was accounted as remainder of the body. Volumes of interest (VOIs) were manually drawn around the organs (slice by slice on the axial plane of CT images) onto each frame of the six acquired scans. Total activity in each organ was determined by multiplying the mean activity concentration (Bq/cm^3^) by the volume of interest. Tissue distribution expressed as percentage-injected dose per organ (%ID/organ) was plotted against time to obtain the time-activity curves (TACs) of measured organs, including the brain and red bone marrow as organs of interest. As for the bone marrow, the estimation of the amount of activity within this tissue was based on the evaluation of the lumbar vertebrae (L1-L5), as described by McParland [11].

### Radiation dosimetry estimates

In this study, radiation absorbed doses and effective doses were calculated based on the RADAR method [12] by entering the time-integrated activity coefficient (formerly known as residence time) of each source organ into OLINDA/EXM 1.1 software (Organ Level Internal Dose Assessment Code, Vanderbilt University, Nashville, USA), using the reference adult male and female models [13]. The OLINDA/EXM kinetic analysis module was used to calculate the time-integrated activity coefficient by applying a three-exponential fit to the six data points, switching ON the option for decay corrected data. Organ volumes derived from CT were converted to mass using density values from the International Commission on Radiological Protection Publication 89 (ICRP-89). Finally, the time-integrated activity coefficients for the gastrointestinal tract were estimated using the ICRP 30 gastrointestinal (GI) tract model included in the OLINDA/EXM code assuming an activity fraction of 0.095 (men) and 0.096 (women) enters the small intestine, as determined from the PET images at 24 h p.i. This model assumes that a fraction of injected activity enters the small intestine with no reabsorption.

### Statistical methods

Data are presented as means ± standard deviation (SD) unless otherwise stated.

## Results

**Fig. 1:**
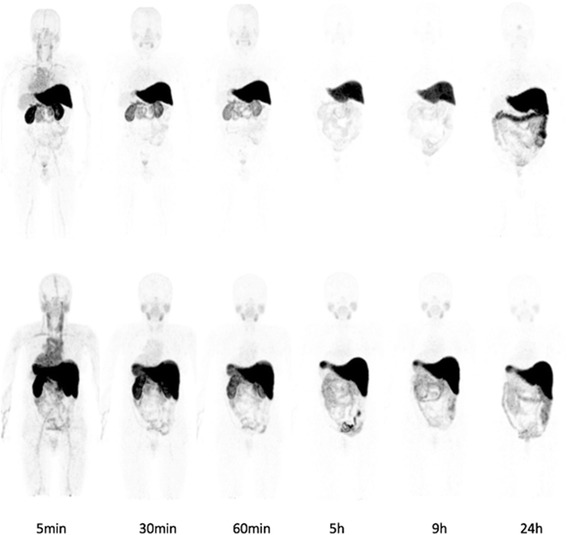
Whole-body PET images (maximum intensity projection) showing typical normal biodistribution of [^64^Cu]CuCl_2_ in males (top row) and females (bottom row) at the specified times post-injection. Injected activity was 4 MBq/kg

**Fig. 2:**
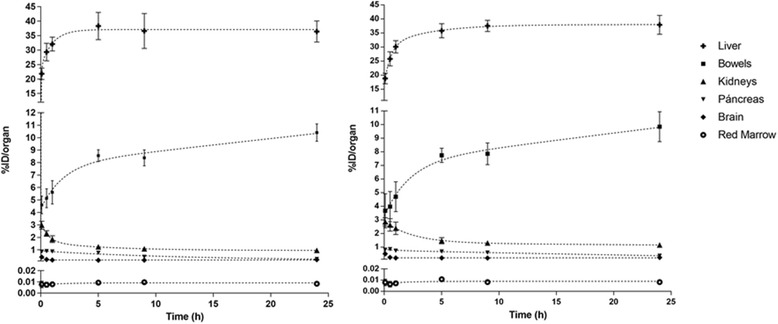
Decay corrected TAC’s of source organs for males (left) and females (right) after injection of [^64^Cu]CuCl_2_

The injection of 310 ± 50 MBq of [^64^Cu]CuCl_2_ solution in physiological saline produced no observable adverse events or clinically detectable pharmacologic effects in any of the six subjects, and no apparent changes in standard vital signs. Given the injected activity and the apparent molar activity of the [^64^Cu]CuCl_2_ used in this research, the administered copper was in trace amount, on the order of nanomoles, which fall well below the known toxicity limit for copper ions. Figure 1 shows typical normal biodistributions of the radiotracer at the evaluated times after injection. Biodistribution data, expressed as %ID/organ, are shown in Fig. 2 for the liver, pancreas, kidneys, bowels, brain, and bone red marrow. The predominant uptake was observed in the liver, reaching a maximum value of (%ID/organ, mean ± SD) 38.3 ± 4.7 at 5 h p.i. for men and 37.9 ± 3.4 at 24 h p.i. for women, followed by the bowel with a maximum uptake of 10.4 ± 2.7 and 9.8 ± 1.3 at 24 h p.i. for men and women, respectively. The time-integrated activity coefficients for the different organs in men and women are listed in Table 1. The mean organ doses and WB effective doses normalized to the unit-injected activity are given in Table 2. The estimated absorbed doses to the critical organ (liver) were 310 ± 67 and 421 ± 56 μGy/MBq for men and women, respectively, whereas the effective doses were 51.2 ± 3.0 and 61.8 ± 5.2 μSv/MBq.

**Table 1:** Time-integrated activity coefficients of source organs (mean ± SD) for healthy subjects injected with [^64^Cu]CuCl_2_

Source organ	Adult male MBq-h/MBq	SD	Adult female MBq-h/MBq	SD
Brain	3.43E-02	3.18E-03	3.90E-02	1.17E-02
Red marrow	1.37E-03	8.13E-04	1.40E-03	4.67E-04
Liver	6.52E + 00	1.45E + 00	6.62E + 00	9.05E-01
Pancreas	7.96E-02	9.12E-03	9.02E-02	1.41E-04
Kidneys	2.06E-01	5.52E-02	2.49E-01	6.93E-02
LLI	5.01E-01	1.38E-01	4.78E-01	1.01E-01
SI	3.30E-01	9.08E-02	3.14E-01	6.62E-02
ULI	6.27E-01	1.73E-01	5.98E-01	1.26E-01
Remainder	9.75E + 00	1.34E + 00	9.57E + 00	1.35E + 00

Abbreviations: *LLI* lower large intestine, *SI* small intestine, *ULI* upper large intestine

**Table 2:** Mean organ absorbed radiation doses and effective doses normalized to the unit-injected activity (mean ± SD) of [^64^Cu]CuCl_2_ in healthy subjects

Organ	Adult Male Absorbed dose (μGy/MBq)	Adult Female Absorbed dose (μGy/MBq)
Adrenals	30.0 ± 0.9	37.8 ± 0.7
Brain	4.2 ± 0.1	5.4 ± 0.5
Breasts	16.0 ± 1.3	20.3 ± 1.9
Gallbladder Wall	44.3 ± 2.9	54.1 ± 2.6
LLI Wall	153 ± 40	161 ± 26
Small Intestine	55.0 ± 11.0	62.6 ± 6.4
Stomach Wall	22.7 ± 1.3	29.1 ± 1.3
ULI Wall	131 ± 31	144 ± 23
Heart Wall	22.9 ± 0.6	29.8 ± 1.4
Kidneys	69.7 ± 15.7	90.0 ± 21.2
Liver	310 ± 67	421 ± 56
Lungs	20.8 ± 0.7	27.2 ± 1.4
Muscle	18.0 ± 1.6	22.5 ± 1.9
Ovaries^*^	24.3 ± 3.8	30.0 ± 0.8
Pancreas	83.4 ± 9.1	105 ± 14
Red Marrow	16.7 ± 1.3	19.9 ± 1.2
Osteogenic cells	32.9 ± 3.8	43.4 ± 5.0
Skin	14.6 ± 1.6	18.3 ± 1.9
Spleen	19.6 ± 1.5	25.3 ± 4.7
Testes	15.5 ± 2.2	–
Thymus	17.3 ± 1.6	22.0 ± 2.3
Thyroid	15.5 ± 2.0	18.8 ± 2.5
Urinary Bladder Wall	18.8 ± 2.6	21.4 ± 2.0
Uterus^*^	21.2 ± 3.0	26.3 ± 1.6
Total Body	26.9 ± 0.1	34.1 ± 0.2
Effective Dose (μSv/MBq)	51.2 ± 3.0	61.8 ± 5.2

^*^Dosimetric calculations for men

## Discussion

This study represents, to the best of our knowledge, the first biodistribution and radiation dosimetry measurements of ^64^Cu in the chemical form of copper dichloride in healthy volunteers. The evaluated [^64^Cu]CuCl_2_ data revealed a biodistribution dominated by activity in the liver, bowels, pancreas, and kidneys. Urinary excretion was negligible. Most fecal copper results from biliary excretion, as the bile is the major pathway for the excretion of copper [14]. The dosimetry calculations revealed the liver as the critical organ with a mean (all subjects, men and women) absorbed dose of 366 ± 87 μGy/MBq. On the other hand, the mean WB effective dose was 56.5 ± 6.0 μSv/MBq. Note that in general the radiation doses are higher for females than males; this is due to the smaller body and organ sizes than those of males. Additionally, female gonads are inside the body instead of outside, receiving a higher radiation dose given the proximity to several organs, such as liver and kidneys, which are often important source organs.

We recently reported the biodistribution of [^64^Cu]CuCl_2_ in healthy rats and estimated radiation doses to humans by extrapolating the animal data [15]. As discussed in our previous publication, these results cannot be directly compared because the biodistribution data were obtained from different species, and the discrepancies in the results most probably reflect the differences among the species in size and metabolic rate as well as differences in the assumptions in the dose calculations [16]. However, what is clear is that the extrapolation of animal (mice and rats) data to humans’ incorrectly identify the critical organ, and underestimate radiation absorbed doses for [^64^Cu]CuCl_2_. Radiation dosimetry estimates of [^64^Cu]CuCl_2_ using biodistribution data from patients with prostate cancer [8] are also underestimated compared to dose estimates based on biodistribution data obtained from healthy volunteers. The difference of radiation-absorbed doses to specific organs and effective doses presented in Table 3 could be explained in part by the altered biodistribution of [^64^Cu]CuCl_2_ in prostate cancer patients, as it has been demonstrated that the biodistribution of ^64^Cu in tumor-bearing animals is altered as compared with that of the corresponding control animals [17, 18], but given the subjectivity of the process of curve fitting, volume drawing, and assumptions in the dose calculations, the differences between our results and those previously reported by Capasso et al. are most likely not just due to altered biodistribution but due to the analysis technique. However, these details are not given in the publication of Capasso et al.

**Table 3:** Comparison of radiation absorbed dose (μGy/MBq) to specific organs and effective dose obtained with biodistribution data from patients suffering prostate cancer [8] and healthy volunteers (this work), after the administration of [^64^Cu]CuCl_2_

	Capasso et al. 2015 [8]	This study		
	Male	Male	Female	Mean (Male & Female)
Liver	294	310 ± 67	421 ± 56	366 ± 87
LLI	19.2	153 ± 40	161 ± 26	159 ± 48
ULI	23.8	131 ± 31	144 ± 23	138 ± 39
Pancreas	28.8	84.3 ± 9.1	105 ± 14	94.7 ± 16.7
Kidneys	33.6	69.7 ± 15.7	90.0 ± 21.2	79.9 ± 26.4
Effective dose (μSv/MBq)	33.8	51.2 ± 3.0	61.8 ± 5.2	56.5 ± 6.0

The most important feature of targeted radionuclide therapy is to deliver therapeutic doses to a tumor, without unduly affecting healthy organs. For most organs, the maximum tolerated dose (MTD) to radiation is on the order of some 10s of Gy, whereas the MTD for the gonads and red bone marrow are as low as 1–2 Gy. It remains to be further investigated whether ^64^Cu in the chemical form of CuCl_2_ will be used as a theranostic agent for the treatment of tumors; however, dosimetry estimations from human distribution data suggest that critical organs such as liver, kidney, pancreas, and intestines, would allow the administration of therapeutic activities of ^64^Cu, on the order of several GBq (hundreds of mCi), without jeopardizing the function of these organs as shown in Table 4. The same applies to other radiosensitive organs and tissues such as gonads and red bone marrow. Note from Table 4 that the effective doses of [^64^Cu]CuCl_2_ are similar to those estimated for ^177^Lu-PSMA, a typical agent used in TRNT [19]. However, the absorbed doses to normal organs are considerably higher for ^177^Lu-PSMA than those of [^64^Cu]CuCl_2_. ^64^Cu is an ideal therapeutic radionuclide given its shorter half-life compared to ^177^Lu and ^90^Y typically used in TRNT applications. The shorter half-life of ^64^Cu allows the injection of higher initial activities to deliver an equivalent absorbed dose to tumors; however, the damage that is done from that equivalent absorbed dose will be greater since the higher activity will result in a greater number of disintegrations, and as such, greater probability of DNA hits and irreparable DNA breakages. Additionally, ^64^Cu can be efficiently produced in Curie quantities in compact biomedical cyclotrons which is far more convenient than the production of ^177^Lu and ^90^Y in nuclear reactors.

**Table 4:** Effective doses for whole body in mSv and absorbed doses for normal organs in Gy after the administration of varying therapeutic activities of [^64^Cu]CuCl_2_ and ^177^Lu-PSMA

Estimations for [^64^Cu]CuCl_2_ (This work)						
Activity (GBq)	Whole body	Liver	Kidney	Pancreas	Lower large intestine	Upper large intestine
4.0	226±24	1.46±0.35	0.32±0.11	0.38±0.07	0.64±0.19	0.55±0.16
8.0	452±48	2.93±0.70	0.64±0.21	0.76±0.13	1.27±0.38	1.10±0.31
12.0	678±72	4.39±1.04	0.96±0.32	1.14±0.20	1.91±0.56	1.66±0.47
Estimations for ^177^Lu-PSMA (Okamoto et al. 2017 [19])						
Activity (GBq)	Whole body	Liver	Kidney	Parotic glands	Submandibular glands	Lacrimal glands
4.0	240±12	0.48±0.24	2.88±0.84	2.2±0.6	2.6±1.60	15.2±6.6
8.0	480±24	0.96±0.48	5.76±1.68	4.4±1.1	5.1±3.2	30.4±11.2
12.0	720±36	1.44±0.72	8.64±2.52	6.6±1.7	7.7±4.8	45.6±16.8

## Conclusions

We have evaluated the biodistribution and dosimetry of [^64^Cu]CuCl_2_ in six healthy adults. These results provide the first human dosimetry data of [^64^Cu]CuCl_2_ in healthy volunteers. In all cases, the liver had the highest dose among all the organs and is deemed to be the critical organ, with a mean absorbed dose of 366 ± 87 μGy/MBq. The mean WB effective dose was 56.5 ± 6.0 μSv/MBq.

## Abbreviations

%ID/organ: Percentage-injected dose per organ
CuCl_2_: Copper dichloride
DICOM: Digital Imaging and Communications in Medicine
hCTR1: Human copper transporter 1
ICRP: International Commission on Radiological Protection
LLI: Lower large intestine
MTD: Maximum tolerated dose
OLINDA: Organ Level Internal Dose Assessment
OSEM2D: 2-D ordered subset expectation maximization
p.i.: Post-injection
p-NH2-Bn-NOTA: 2-S-(4-Aminobenzyl)-1,4,7-triazacyclononane-1,4,7-triacetic-acid
SD: Standard deviation
SI: Small intestine
TAC: Time-activity curve
TRNT: Targeted radionuclide therapy
ULI: Upper large intestine
VOI: Volume of interest
WB: Whole-body
